# Diffusion weighted MR imaging in acute vertebral compression fractures: differentiation between malignant and benign causes

**DOI:** 10.2349/biij.2.2.e12

**Published:** 2006-04-01

**Authors:** AA Bhugaloo, BJJ Abdullah, YS Siow, KH Ng

**Affiliations:** 1 Department of Biomedical Imaging, Faculty of Medicine, University of Malaya, Kuala Lumpur, Malaysia; 2 Department of Orthopaedic Surgery, Faculty of Medicine, University of Malaya, Kuala Lumpur, Malaysia

**Keywords:** Diffusion weighted imaging, MRI, metastases, vertebral fractures

## Abstract

**Aim:**

The primary objective of this study was to evaluate the specificity and sensitivity of diffusion weighted MR imaging (DWI) in the differentiation and characterisation between benign and malignant vertebral compression fractures compared with conventional T1 WI, T2 WI and fat suppressed contrast enhanced T1 WI in the Malaysian population.

**Materials and Methods:**

Thirty five patients with 68 vertebral compression fractures were imaged using the conventional T1 WI, T2 WI, fat suppressed contrast enhanced T1-weighted, and steady state free precession diffusion-weighted (SSFP DWI) sequences on a 1.5 T MR scanner. Signal intensities were analysed qualitatively for all the sequences by comparison to adjacent normal marrow. A quantitative assessment of the signal intensity in the SSFP DWI was also performed.

**Results:**

T1 WI and T2 WI images are of limited diagnostic value because of the variability in signal intensities. Contrast enhanced images had sensitivity and specificity of 93% and 71%, respectively with a negative predictive value (NPV) of 93%. On diffusion-weighted MR imaging, sensitivity was 87% with specificity of 92%. The positive predicative value (PPV) and NPV were both 90%. The quantitative assessment of ratio revealed a statistical significant difference between the benign (0.96) and the malignant (1.73) group of lesion (Mann-Whitney U-test, *p*=0.0001).

**Conclusions:**

We found that absence of contrast enhancement has a high NPV (90%) while SSFP DWI has both a high PPV (90%) and high NPV (90%) in detecting malignant vertebral compression fractures. Furthermore, in our study the ratio of lesion intensity technique offers an excellent criterion to differentiate between the benign and malignant lesions, and the presence of iso- or hypointensity of the collapsed vertebral bodies is suggestive of a benign lesion while hyperintensity is highly suggestive of malignancy. We also found that using the NLMR showed a statistical significant difference between the malignant and benign groups (*p*<0.0001) with osteoporotic and malignant lesions have mean values of 0.96 (SD 0.25) and 1.73 (SD 0.4) respectively.

## INTRODUCTION

Vertebral fractures may be detected on radiographs, computed tomography or radionuclide studies, but in today’s clinical environment, the specific discrimination between benign and malignant vertebral compression fractures relies heavily on MR imaging features. Since most bony metastases are hematogeneous in origin, the axial skeleton is the most common site of skeletal metastases initially due to abundant vascularisation and red bone marrow [1]. However, osteoporotic compression fractures are also a common occurrence in the spine and can be confused with metastatic compression fracture in the acute phase. Since the prognosis and management differs in these two entities, accurate diagnosis is important.

The primary objective of this study was to evaluate the specificity and sensitivity of diffusion weighted MR imaging (DWI) in the differentiation and characterisation between benign and malignant vertebral compression fractures compared with conventional T1 WI, T2 WI and fat suppressed contrast enhanced T1 WI in the Malaysian population.

## MATERIALS AND METHODS

The study was carried out prospectively from July 2002 to June 2004. Thirty five consecutive patients with a history of vertebral compression fracture detected by other imaging modalities were included. Selected patients were imaged within six weeks from the time of presentation. Patients with history of vertebral compression fracture of more than six weeks, patients who were not MR compatible as well as patients who had vertebral collapse secondary to disciitis or osteomyelitis (as they had other features to suggest their diagnosis e.g. paravertebral enhancing collection and/or loss of disc spaces with erosion of the endplates) and those with sclerotic lesions were excluded from the study. Patients with vertebral fractures secondary to severe trauma were also excluded.

The study group consisted of 13 men and 22 women, ranging from 25 to 88 years, with a mean age of 62.7 (SD 14.2) and a median age of 64.5. The 68 vertebral compression fractures were noted. The patients were imaged using the conventional T1 WI, T2 WI, fat suppressed contrast enhanced T1-weighted, and steady state free precession diffusion-weighted (SSFP DWI) sequences [2] (Table 1) using a spinal phased array coil on a 1.5 Tesla super conducting MR System (Magnetom Vision, Siemens, Erlangen, Germany). The SSFP DWI sequence used 18 NEX with a diffusion pulse length of 2 ms. The diffusion gradient was 24mT/m with a relatively low b value (165s/mm2). The diffusion gradient was applied only in the readout direction based on the previous observation that no diffusion anisotropy was found in either the phase or slice direction [3]. Additional axial views were obtained only in those cases where the marrow changes were focal.

**Table 1:** Scan parameters for the MR sequences

	**T1**	**T2**	**C.E**	**SSFP DWI**
TR (sec)	587	4000	600	0.0216
TE (sec)	12	128	12	0.005
Thickness (mm)	4	4	4	4
Orientation	Sagittal	Sagittal	Sagittal	Sagittal
Matrix	366x512	276x512	126x256	168x256
FOV (mm)	300	280	280	250
Pixel Size (mm)	0.6x0.6	1.0x0.5	1.1x1.1	1.3x0.98
Scan Time	5min 4sec	3min 4sec	3min 58sec	1 min 49 sec

The SSFP sequence was chosen as the other types of DWI (Spin Echo and EPI) were not available on our system. In addition, SSFP MR Diffusion technique has relatively good image quality, SNR and Contrast to Noise Ratios and can be acquired with a relatively short acquisition time (approx. 1 min and 49 seconds in our study) with moderate gradient strengths. Also, there have been numerous studies using this sequence and would therefore be more comparable with the work of others.

The 68 lesions were distributed from the sixth cervical to the fifth lumbar vertebral bodies with most occurring in the T10 to L2 vertebral bodies (50/68). The medical records of these patients were reviewed (BJJA, AAB, YSS) to document the final diagnosis based on either or both clinical and histopathological grounds.

Of the 68 lesions in 35 patients, 38 lesions were established as being benign, while the remaining 30 were categorised as malignant. With regards to the metastases: seven of the lesions were from a sarcoma, four from lung carcinoma, and two each from nasopharyngeal carcinoma, breast carcinoma, hepatocellular carcinoma, renal cell carcinoma and Non-Hodgkin lymphoma. In addition, four of collapsed vertebral bodies were due to multiple myeloma.

The images obtained were analysed both qualitatively and quantitatively. For qualitative evaluation, the images were analysed and categorised by two experienced radiologists (BJJA, AAB) independently and then in a consensus review. The lesions were characterised as focal or multiple, with or without involvement of the vertebral elements. The signal intensities of the fractured vertebra were visually compared with that of the presumed normal vertebra on all (T1 WI, T2 WI, fat suppressed contrast [CE] enhanced T1-weighted and DWI) and categorised as hypointense, isointense or hyperintense relative to the areas of presumed normal marrow. Statistical evaluation of the qualitative analysis between the two groups was performed using the Mann-Whitney U test.

For the quantitative assessment, the SSFP DWI MR images were analysed with the aid of the OSIRIS software versions 4.19 (Geneva, Switzerland). Signal intensity in the fractured vertebra was quantified by placing a ROI over the lesion. The size of the ROI used occupied at least three quarters of the area of abnormal or normal signal intensity but excluding the end-plates, cortical margins, disc spaces or adjacent normal or abnormal marrow. The abnormal marrow was that seen completely in the selected sagittal DWI images (to reduce partial voluming) while for normal adjacent marrow all the sequences were evaluated (T1, T2, CE MRI and DWI) to ensure that there were no signal abnormalities within the vertebral body selected. The ROI was placed at approximately the same distance from the posterior part of the vertebral body. This was to compensate for any differences in signal intensity as a result of image normalisation. A ratio of the quantified signal intensity of the collapsed vertebra to the presumed normal vertebral bone marrow [termed Normalised Lesion to Normal Marrow (NLNM) ratio] was then calculated and normalised to the normal marrow as follows: [SI (abnormal marrow) – SI (normal marrow)]/ SI (normal marrow)].

The mean value of the NLNM ratio was calculated for each category of compression fracture. A composite histogram was produced for all the patients in the benign and the malignant group of fracture. A box plot of NLNM ratio was also obtained for the benign and the malignant group of compression fractures which shows a maximum and minimum value, together with a mean value. Statistical analysis was performed by using Student’s *t-*test. A *p* value of less than 0.05 was considered a statistically significant difference.

## RESULTS

### Significance of focal or multiple lesions

Eighteen patients had a single lesion, while 17 patients had two or more lesions (there were seven patients who had more than two lesions). Of those with a single lesion, only 44% (8/18) had malignant compression fractures with the remaining 56% (10/19) having osteoporotic fractures. For those with more than one lesion, 59% (10/17) were diagnosed as having malignant compression fracture while 41% (7/17) had osteoporotic compression fractures.

### Role of conventional T1 and T2 weighted imaging

All the affected vertebrae in the benign group and 29 of the 30 lesions in malignant group of compression fractures were either isointense or hypointense with respect to the presumed normal marrow on the T1 W images (Table 2). On T2 WI, 50% (15/30) of the malignant lesions were either iso- or hypointense, while the remainder (15/30) were hyperintense. Only 13% (5/38) of osteoporotic compression fractures showed high signal intensity on the T2 weighted images.

**Table 2:** Signal Intensity of the fractures on T1, T2, Contrast Enhancement and DWI

**Sequence**	**T1**			**T2**			**Contrast Enhanced**		**DWI**		
Established Diagnosis	Iso	Low	High	Iso	Low	High	Enhanced	Not Enhanced	Iso	Low	High
Osteoporotic Fracture	13 (34%)	25 (66%)	0	25 (66%)	8 (21%)	5 (13%)	11 (29%)	27 (71%)	9 (24%)	26 (68%)	3 (8%)
Malignant Fracture	12 (40%)	17 (57%)	1 (3%)	10 (33%)	5 (17%)	15 (50%)	28 (93%)	2 (7%)	0	4 (13%)	26 (87%)

With regards to changes of the end plates and disc spaces, these changes were seen in 11% (4/38) and 10% (3/30) of the osteoporotic and malignant groups respectively. Posterior element involvement was seen in 23% (7/30) of lesions in the malignant group but in none of the osteoporotic lesions (0/38). Paraspinal masses were seen in 5% (2/38) and 23% (7/30) of the osteoporotic and malignant groups respectively. Cord compression was noted in 27% (8/30) of the malignant and 11% (4/38) of the osteoporotic groups.

### Contrast enhanced images

On contrast enhanced imaging, 93% (28/30) of the malignant and 29% (11/38) of the osteoporotic compression fractures showed enhancement (Table 2). The sensitivity and specificity of contrast enhancement for malignant vertebral compression fractures was 93% and 71% respectively. The positive predictive value of contrast enhancement was 71% (28/39) while the negative predictive value was 93% (27/29) i.e. the absence of enhancement makes the likelihood of benign fractures high.

### Qualitative analysis of DWI MR imaging

**Figure 1:**
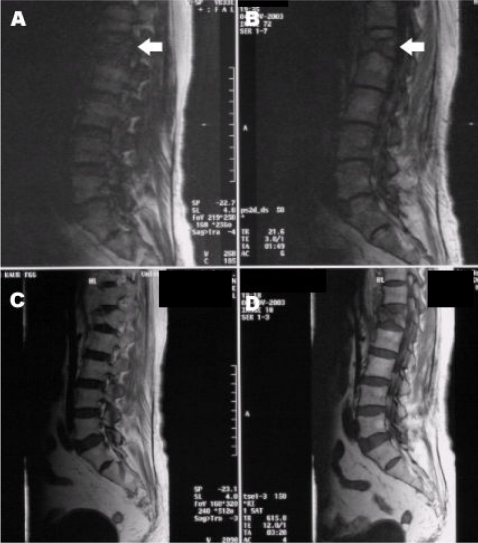
Mid sagittal MR images of the spine with an osteoporotic fracture of L1 vertebral body. Mid sagittal SSFP DWI MRI at a TE of 5ms (A) and 3ms (B) show low signal intensity (arrow) in the collapsed L1 vertebral body compared to the normal bone marrow in adjacent vertebral bodies. There is no contrast enhancement of the L1 vertebra (C) after gadolinium administration.T1 W MR image (D) shows low signal in the L1 vertebra.

**Figure 2:**
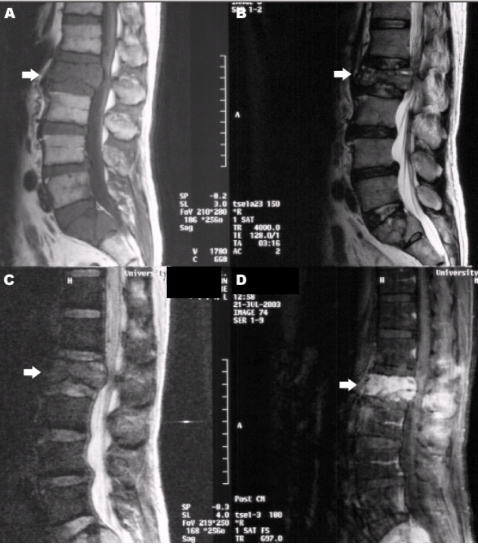
MR images of a patient who presented with compression fracture of L2 vertebral body. There are low signals on both T1 (A) and T2 (B)-weighted MR images, with marked contrast enhancement (D) of L2 and S1 vertebral bodies. SSFP DW MR image shows high signal in the compressed L2 (arrow) as well as in S1 vertebral bodies suggestive of malignancy. Further investigation revealed a bronchogenic carcinoma.

The signal intensities of the osteoporotic vertebral fractures on the DWI was low in 68% (26/38) of lesions (Figure 1), isointense in 24% (9/38) of lesions and hyperintense in only 8% (3/38) of lesions (Table 2). In the malignant group, the fractured vertebral bodies were hyperintense in 87% (26/30) of lesions (Figure 2) and hypointense in 13% (4/30) of cases, while there were no lesions which were isointense. Using the presence of high signal intensity on DWI as indicator of malignant disease, the sensitivity and specificity of DWI was 87% and 92% respectively. Of the 26 malignant lesions which were high signal on DWI, only 6 were high signal on T2 WI with 10 each being iso-intense and low signal. The positive predictive value of high signal on DWI for malignant fractures was 90% (26/29) while the negative predictive value was also 90% (35/39). There were 2 lesions in one patient which showed contrast enhancement even though the DWI signal was low.

### Quantitative analysis of DWI MR imaging

**Figure 3:**
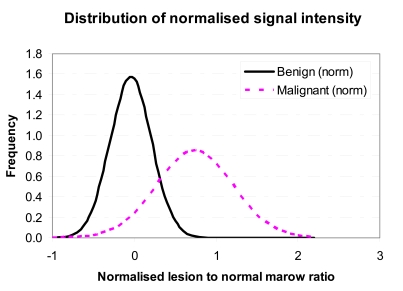
Frequency distribution of the normalised ratio of lesion to normal marrow signal intensity of malignant and benign vertebral compression fracture on DWI.

**Figure 4:**
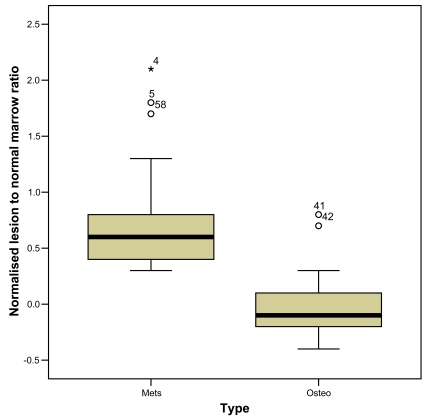
Box and whisker plot of the normalised ratio of lesion to normal marrow signal intensity of benign and malignant compression fracture. The boxes show minimum and maximum values for each category and the whiskers indicate the =/- 2SD. Those values beyond these (the outliers) are plotted but excluded from the calculation of SD. The mean ratio, as indicated by a horizontal line in the box, is 0.9 for the benign group of lesion and 1.65 for the malignant group. The mean value is significantly different for the two categories and there is no significant overlap of values between the two boxes.

A frequency distribution was produced for both the benign and the malignant group of vertebral compression fracture (Figure 3). The Normalised Lesion to Normal Marrow (NLNM) ratio for the benign group of compression fractures revealed a mean of -0.04 with a SD of +0.25 while that for the malignant group was 0.74 with a SD of +0.47. A box plot of NLNM ratio (Figure 4) was also obtained for each group of lesion. The quantitative assessment revealed a statistical significant difference between the two groups of lesions, (Mann-Whitney U-test, *p*=0.0001).

## DISCUSSION

Benign vertebral lesions occur in approximately one third of cancer patients [4] while metastatic vertebral lesions account for 39% of bony metastases in patients with primary neoplasm [5]. Differentiation between malignant and benign vertebral compression fracture is a common problem in medicine. This is especially so in the elderly patients who are predisposed to benign compression fracture caused by osteoporosis, where establishing the correct diagnosis is of great importance in determining treatment, surgical approach, and prognosis [6,7]. In this group a benign fracture can result from minor trauma and make the interpretation of the lesion difficult if there is a known primary elsewhere.

Although MR imaging using conventional T1 WI and T2 WI has proved helpful in differentiating between benign and malignant causes of vertebral collapse, confident diagnosis is not always possible. Morphologic signs such as the degree and pattern of bone marrow replacement, multiplicity of lesions, paravertebral soft-tissue masses, infiltration of posterior elements of the vertebrae [7-10] and presence of a fracture line in osteoporotic fractures (as a linear hypointensity in the middle of the compressed vertebral body or adjacent to a compressed endplate) usually seen on T2- or post-contrast T1-weighted images [10,11] are common signs used for assessing the cause of the fracture. Despite the use of these features, there is still considerable overlap in the signal changes between acute to sub-acute fractures from malignant fractures as was also found in this study [12,13]. The presence of multiple collapsed vertebrae also does not suggest a benign or malignant aetiology. In our study it was found that 59% of patients with malignant involvement had multiple level involvements while this was seen in 41% of those with osteoporotic fractures.

CE is used to identify intramedullary spinal cord abnormalities and extradural lesions (particularly in the epidural space) that may result in compression of the spinal cord and alter proposed treatment, however this has not been assessed to determine the underlying aetiology. Benign vertebral fractures may also enhance after intravenous administration of contrast media due to a breach in blood tissue. We found that using contrast enhancement with fat suppression as an indicator of malignancy; the sensitivity was 93%, the specificity was lower at only 71% while the negative predictive value was found to be 93%. Even though dynamic contrast enhancement has been evaluated in the characterisation of lesions in the brain, liver breast, pelvis, etc, [14] this has not been evaluated in the spine.

Over the last decade, DWI MR imaging [15-17] of the vertebral body [18] has received considerable attention and has been successfully implemented for the differentiation of benign and malignant fracture oedema (due to tumour infiltration) [19,20] although the usefulness is still controversial [5,21]. DWI MRI provides unique tissue characterisation that is complementary to that provided by conventional MR Imaging and is sensitive to micro-structural changes. The reduced mobility of water in pathologic fracture is the result of tumour cell accumulation and subsequent reduction in the interstitial spaces that results in high signal intensity compared with normal bone marrow. On the other hand, the increased mobility of water attributed to an increase in the interstitial space in relation to oedema or haemorrhage [19] in benign fractures [20,22-24] results in low signal intensity in benign osteoporotic and traumatic fractures. On this basis DWI MRI has been suggested to be useful particularly in the evaluation of vertebral lesions.

Bauer *et al.* [3] found 100% accuracy in the diagnosis of malignant compression fractures using SSFP DWI. They also showed that even though T1 Weighted spin echo and T2 Weighted STIR scans detected all fractures, there was no discriminating power based on signal intensity or bone marrow contrast ratio. In our study, we found that the SSFP DWI sequences showed a high diagnostic accuracy in differentiating acute benign osteoporotic fracture from pathological fractures with a sensitivity of 87%, a specificity of 92% with a PPV of 90%. Even though the sensitivity of contrast enhanced MRI was higher at 93%, the PPV was only 71%. We found that the NPV for low or iso-intense on DWI was 90% for acute benign fractures.

Even though some studies [25] have demonstrated no advantage of diffusion weighted scanning in the detection or characterisation of vertebral metastases with only 34% being hyperintense on DWI, it has been pointed out [26] that the patients enrolled were not the primary target group for DWI in spine (i.e., patients with sclerotic metastases and previously treated metastases). It has been suggested that the following inclusion criteria be used for DWI: 1) unknown reason for the vertebral collapse, 2) lack of sclerotic metastases, and 3) no prior therapy. None of our patients had any prior therapy and those with sclerotic lesions were also excluded.

It has been suggested [25] that T2 “shine through” may be playing a prominent role in the appearance of the metastatic lesions on DWI and that all the metastatic lesions that were hyperintense on DWI MRI are also hyperintense on T2 WI. However, in our study we found that of the 26 malignant lesions which were high signal on DWI, only 23% (6/26) were high signal on T2 WI suggesting that T2 shine through may not be the cause of increased signal on FFSP DWI. We are unable to explain this difference, hence a study with a larger sample size is suggested.

DWI appears to be reproducible with diverse diffusion weighted MR techniques e.g. Spin Echo DWI, the Echo Planar DWI, and the Steady State Free Precession DWI (SSFP) for the differentiation of benign from malignant acute vertebral fractures [18]. Both the spin echo (SE) and the stimulated echo (STE) sequences have played a pioneering role in DWI with relatively high signal-to-noise ratio (SNR) and lower sensitivity with respect to homogeneities in the susceptibility of the measured object. However, these sequences require long acquisition times and with significant “ghosting” artifacts. Echo planar Imaging DWI can be performed within a few seconds, reduces motion artifacts and allows calculation of the ADC value though with a lower signal to noise ratio and is prone to susceptibility artifacts. It is therefore limited in musculoskeletal imaging. SSFP [3] diffusion techniques show relatively good image quality, signal to noise (SNR) and Contrast to Noise Ratios and can be acquired with a relatively short acquisition time (approx. 1 min and 49 seconds in our study) with moderate gradient strengths. However, due to its complicated signal generation and its T1 and T2 dependence, precise ADC and b values cannot be calculated. The b value for different diffusion pulse length can only be approximated from phantom measurements with known T1 and T2 parameters [21].

The Apparent Diffusion coefficient (ADC) expresses the diffusion of water protons in a region of interest and is calculated by a regression analysis of the signal and overcomes the confounding relaxation phenomena, so-called T2 shine-through effects, and perfusion effects that may mask diffusion related SI patterns [20]. The ADC of normal vertebrae is significantly higher than that of vertebral metastases and it is proposed that ADC is a dependable and quantifiable parameter with which to distinguish metastases [27] and it might be used to monitor treatment by revealing treatment-related changes in tissue characteristics [28]. We were unable to determine the ADC values due to the SSFP DWI sequence chosen.

DWI MRI also has a role in other types of tissues in the musculoskeletal system; DWI may allow differentiation of viable and necrotic tumour tissue [28], to study the diffusion in cartilages [29] and joint effusions from degenerative osteoarthritis, inflammation or trauma [30], monitor the response to medical therapy of metastatic spine disease [2], allow delineation of acute spinal cord ischemia, determine structural integrity of the spinal cord, improved detection of ischemic lesions, clarification of the relationship between clinical disability and structural damage to the cord (including degenerative disorders), and monitoring of anti-inflammatory or neuroprotective therapies [18].

We were not able to confirm the histological diagnosis in all our patients as it was not possible to obtain consent for biopsies especially in those with a diagnosis of osteoporotic fractures. But with follow-up of those without biopsies we were able to overcome this limitation. We were unable to quantify the isotropic diffusion coefficient since we used a SSFP DWI sequence and therefore were not able to quantify the ADC. In addition, DWI SSFP may exhibit hyperintensity in infectious disease similar to tumourous fracture in vertebral bodies [31,32] while false Negativity may be accounted for by previous radiotherapy (due to necrosis as compared with viable tumour) or due to excessive fibrosis and bleeding. Moreover, the signal intensity on DWI MR Images depends on the b factor, which is strongly influenced by hardware components, imaging parameters and the pulse sequence itself [18]. This limits comparison between subsequent investigations, for example, follow up studies and monitoring.

When the findings on routine MR sequences are not completely conclusive for the diagnosis of acute benign or malignant vertebral body compression fracture, then the use of both contrast enhancement and diffusion weighted MR sequence may be helpful. We found that absence of contrast enhancement has a high NPV (90%) while SSFP DWI has both a high PPV (90%) and high NPV (90%) in detecting malignant vertebral compression fractures. Furthermore, in our study the ratio of lesion intensity technique offers an excellent criterion to differentiate between the benign and malignant lesions. At a TE of 5 ms, the presence of iso- or hypointensity of the collapsed vertebral bodies is suggestive of a benign lesion while hyperintensity is highly suggestive of malignancy.

With regards to the use of semi-quantitative diffusion measurement, even though recent studies suggest that DWI intensity values alone are highly unspecific [20,23], we found that using the NLMR showed a statistical significant difference between the malignant and benign groups (*p*<0.0001) with osteoporotic and malignant lesions having mean values of 0.96 (SD 0.25) and 1.73 (SD 0.4) respectively.
